# Postdoctoral T32 training is correlated with obtaining an academic primarily research faculty position

**DOI:** 10.1371/journal.pone.0303792

**Published:** 2024-06-07

**Authors:** Adrienne L. Mueller, Addie Schnirel, Sofie Kleppner, Philip Tsao, Nicholas J. Leeper

**Affiliations:** 1 Stanford Cardiovascular Institute, Stanford University School of Medicine, Stanford, California, United States of America; 2 Office of Postdoctoral Affairs, Stanford University, Stanford, California, United States of America; 3 VA Palo Alto Health Care System, Palo Alto, California, United States of America; 4 Division of Cardiovascular Medicine, Department of Medicine, Stanford University School of Medicine, Stanford, California, United States of America; 5 Division of Vascular Surgery, Department of Surgery, Stanford University School of Medicine, Stanford, California, United States of America; Institute of Medical Biochemistry Leopoldo de Meis (IBqM) - Federal University of Rio de Janeiro (UFRJ), BRAZIL

## Abstract

The mission of NIH-sponsored institutional training programs such as the T32 is to provide strong research and career training for early career scientists. One of the main avenues to pursuing health-related research is becoming research faculty at an academic institution. It is therefore important to know whether these programs are succeeding in this mission, or, if barriers exist that prevent trainees from pursuing these careers. Our institution currently trains ~ 2400 post-doctoral scholars per year, approximately 5% of whom are enrolled in one of our 33 T32 programs. In this study, we 1) compare the proximal professional career trajectories of T32 trainees with non-T32 trainees at our institution, 2) compare proximal career trajectories of trainees in a subset of cardiovascular T32 programs based on their previous training backgrounds, and 3) survey past and current T32 trainees in a subset of cardiovascular T32 programs about the barriers and enablers they experienced to pursuing research-oriented careers. We find that former T32 trainees are significantly more likely to attain appointments as primarily research faculty members, compared to other trainees. Trainees report a perceived lack of stability, the paucity of open positions, and the ‘publish or perish’ mentality of academia as the top barriers to pursuing careers in academia. However, they were still more likely to choose research over clinical careers after participating in a dedicated T32 program. Our results support the conclusion that structured training programs strengthen the pipeline of young scientists pursuing careers in academic research, including those from underrepresented backgrounds. However, T32 postdoctoral researchers are held back from pursuing academic careers by a perceived lack of stability and high competition for faculty positions.

## Introduction

In 2018, the White House released the STEM (Science, Technology, Engineering, and Math) Education Strategic Plan: a federal strategy for providing Americans with persistent, high-quality access to STEM education and to position the US as a global leader in STEM professions [1]. The health of our academic research workforce is under threat as postdocs and graduate students increasingly choose alternative career paths [2–4]. A key component of the government’s initiative is the National Institutes of Health (NIH)’s continued investment in formal training programs for early career researchers. The Ruth L. Kirschstein Institutional National Research Service Award (NRSA) [5], also known as a T32 award, is considered the backbone of its training programs. The main goal of T32 programs is to provide strong research and career training for early career scientists, who will then be leaders in STEM, particularly as research faculty. T32 programs prepare individuals who are committed to a research career to transition to their next career stage. These programs not only provide salary support for post-doctoral trainees, but also typically include a wide range of structured career development programming, including workshops, discussions and Individual Development Plans, to enable trainees’ successful transition to the next stage of their careers. The programs support dedicated research training time in the labs of faculty mentors, but also ensure that all trainees are well-versed in the fundamentals of practicing sound science mandating training in rigorous and reproducible research methods and the responsible conduct of research. T32 training typically lasts two and a maximum of three years, and is not repeatable during a trainee’s postdoctoral training period. It is therefore important to know whether these investments are succeeding in this goal, and what the factors are that contribute to, or inhibit, that success.

An additional goal of NRSA T32 training programs is to increase STEM workforce diversity. US academic professoriates populations have very poor recruitment and retention of underrepresented racial and ethnic minorities [6]. One of the criteria T32 programs are evaluated on is their recruitment plan to enhance diversity and their success in recruiting and supporting trainees from diverse backgrounds. It is therefore also important to know whether NRSA programs are effective in bolstering trainees from underrepresented backgrounds’ participation in academic research.

Our Tier 1 research institution currently hosts 33 distinct T32 training programs spanning all aspects of human health and disease, including three that are specifically related to cardiovascular research. These 33 T32 programs support approximately 5% of our total current post-doctoral trainee population, the remainder of whom do not necessarily participate in the structured programming offered via the T32 mechanisms. These three cardiovascular T32 programs are administered or co-administered by a single organizing unit within the institution, making their data and trainee population readily available for study.

In this study, we compare the proximal career trajectories of T32 trainees with non-T32 trainees at our institution, including trainees who self-identify as underrepresented minorities. We also summarize the surveyed reports of past and current trainees from a subset of high-performing T32 programs in cardiovascular science about the barriers and enablers they experienced to pursuing careers in academia.

## Methods

### Study design and participants

Data for this study comes from responses to three data sources: 1) data assembled by the institutional Office of Postdoctoral Affairs both from institutional records and research on proximal career trajectories of postdoctoral trainees (OPA Data) who completed their training at Stanford between 2010 and 2020; 2) data from three cardiovascular T32 program training records and progress reports (CV T32 Data), and 3) a survey conducted in 2020 by representatives from the three cardiovascular T32 programs on the barriers and enablers that past and current postdoctoral trainees in the training programs experienced in pursuing careers in academia (CV T32 Survey). This survey was deemed exempt from human subject’s research by the Stanford University Institutional Review Board because the only involvement of human subjects in the research activities will be in one or more of the categories that are exempt from the regulations at 45 CFR 46 or 21 CFR 56 (IRB# 58548). See Table 1 for information on the numbers and demographics of individuals in the three datasets (OPA Data, CV T32 Data, CV T32 Survey). Individuals in OPA Data were classified as having confirmed participation in a T32, “T32 Postdocs,” or not, “Non-T32 Postdocs”. For this dataset, only postdocs in relevant health-related departments were included. Note that we were unable to verify the T32 status of all of the postdocs in the “Non-T32 Postdocs” group, so there may still be a small percentage of T32 postdocs in this population (n = 54–108 out of 2,021). Amongst the T32 trainees, the subset who participated in one of our three cardiovascular T32s were also identified “Cardiovascular T32 trainees”. “T32” respondents are individuals who are confirmed to have participated in one of 16 postdoctoral T32 programs at our institution, including the three cardiovascular T32 programs. Participant data was not available for the remaining 17 out of 33 postdoctoral T32 programs. “Non-T32 Postdocs” respondents are individuals for whom their T32 participation status is not confirmed. All 81 cardiovascular T32 trainees were asked to complete the survey. Of the 81 cardiovascular T32 trainees for whom data was available, 49 (60%) responded to the CV T32 Survey.

**Table 1:** Demographics of study datasets: *OPA Data*, *CV T32 Program Data*, and *CV T32 Survey Data*.

OPA Data			CV^a^ T32 Program Data	CV^a^ T32 Survey Data
Sex	T32 Postdocs	Non-T32 Postdocs		
Female	126 (50.8%)	1,049 (51.9%)	39 (48.1%)	24 (49%)
Male	122 (49.2%)	972 (48.1%)	42 (51.9%)	25 (51%
URM^b^ Status				
URM^b^	32 (12.9%)	207 (10.2%)		
Not URM^b^	216 (87.1%)	1,813 (89.7%)		
Unknown	0 (0%)	1 (0%)		
Total	248 (100%)	2,021(100%)	81 (100%)	49 (100%)

^a^CV = cardiovascular

^b^URM = underrepresented minority

### Measures

In the OPA dataset, the careers of former postdocs were identified using web searches to verify employment via employer websites such as University faculty profiles, research databases, and social media sites such as LinkedIn. The careers of former postdoctoral trainees were taxonomized based on a refined version of UCOT 2017 [7] where UCOT 2017 refers to a taxonomy developed by several organizations and described in Silva *et al*, 2019 [8]. This taxonomy was developed and applied in order to compare job sectors and career types consistently across institutions, however adoption is still in the early stages with minimal comparison across institutions available to date. This career taxonomy includes Job Sector (Academia, For-Profit, Government, Nonprofit, or Other) and “Career Type (“Further Training or Education,” “Primarily Research,” “Primarily Teaching,” “Science/Discipline Related,” or “Not Related to Science/Discipline”). Descriptions of these can be found in Silva et al 2019 [8]. Additionally, a faculty flag indicator was used to highlight those holding faculty roles as refined by Stayart et al 2020 [7].

**Fig 1:**
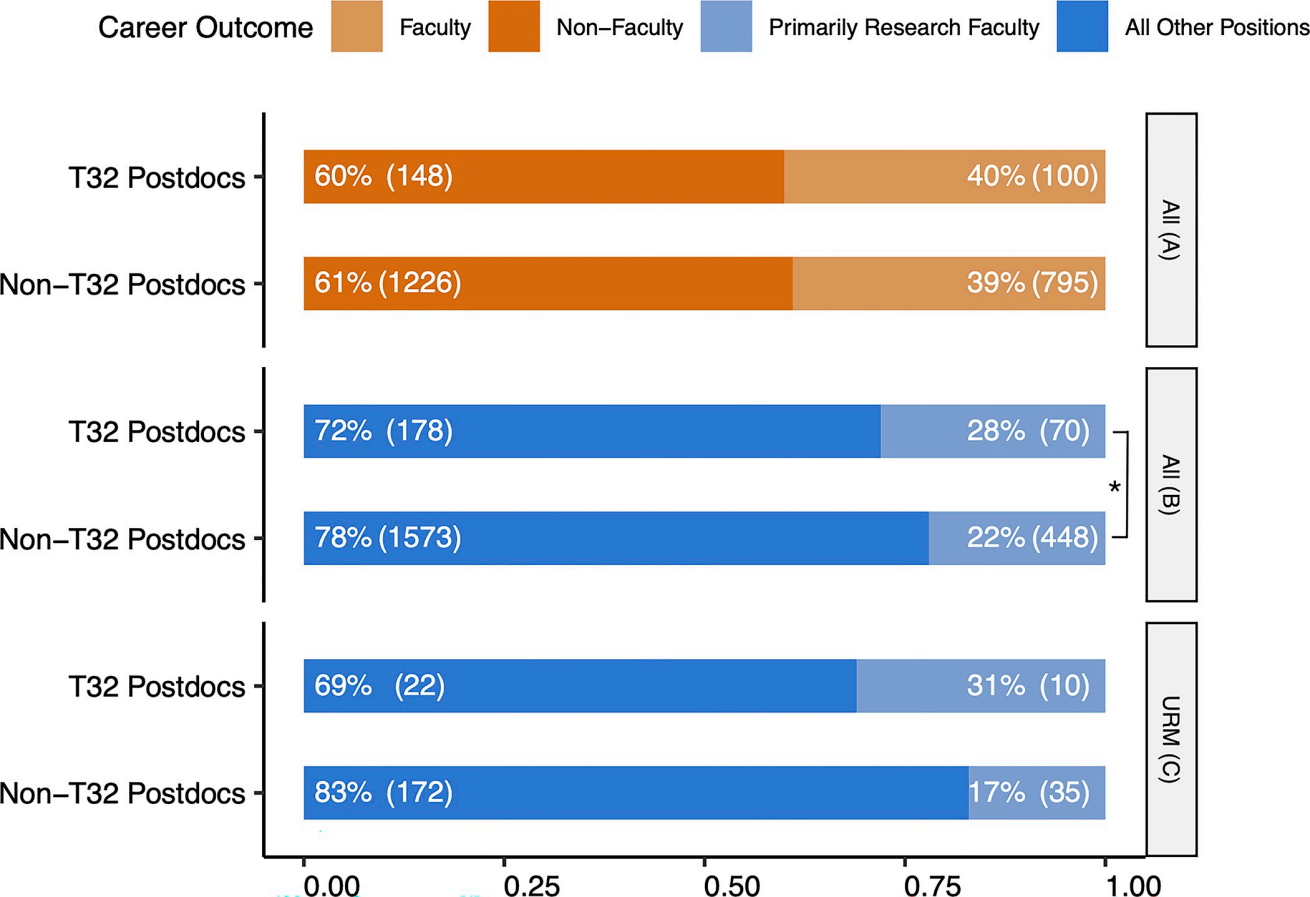
Proximal career trajectories of T32 and non-T32 postdoctoral trainees. **A**: The proportion of trainees identified as holding either faculty or non-faculty positions for two populations: T32 Postdocs (N = 248) and Non-T32 Postdocs (N = 2021). **B**: The proportion of trainees who identified as holding either primarily research faculty positions or any other position for T32 Postdocs (N = 248) and Non-T32 Postdocs (N = 2021). * indicates significance at p < 0.05 level. **C**: Among trainees who identified as underrepresented minorities, the proportion of trainees who identified as holding either primarily research faculty positions or any other position for two populations: trainees with T32 Postdocs (N = 32) or Non-T32 Postdocs (N = 207). Data in panels A, B and C are from the OPA dataset. All = all postdoctoral trainees in the OPA dataset; URM = underrepresented minority postdoctoral trainees in the OPA dataset.

Postdoctoral trainees were grouped in two ways: The first grouping (orange tones in Fig 1) is strictly comparing those who have faculty positions versus those who are not, where the faculty role could be primarily teaching at any college or university. The second grouping (blue tones in Fig 1) identifies those who have “Primarily Research Faculty” positions or not i.e. “All other positions.” If they are identified as faculty, in the sector “Academia”, whose work is “primarily research” (usually at a research-intensive university) then they have a “Primarily Research Faculty” role. Additionally, individuals from this dataset were identified as either underrepresented minorities or not via Stanford University biodemographic data.

OPA collected career taxonomy data for 10,620 former postdocs who completed their Stanford appointments between 2011 and 2019. Since T32 trainees are citizens or permanent residents only, we reduced the entire data set by removing international postdocs. Using only citizens or permanent resident data allows a less-biased comparison between T32 and non-T32 trainee populations, because trainees have similar career and funding opportunities, such as K awards. We further reduced the data set to only include former postdocs from the School of Medicine and the departments of Biology, Materials Science and Engineering, Mechanical Engineering, or Bioengineering. This left 2269 former postdocs who were citizens/permanent residents and in a relevant field.

The proximal career trajectories of T32 trainees in the three participating programs were identified based on information collected for the programs’ 2020 NIH-mandated progress reports. Data in these progress reports include information from all trainees, including current trainees and trainees appointed in previous funding cycles. To collect this information, we either reached out to the trainees directly for information about their current career status or identified their current occupation through online search. Trainees’ current career trajectories were categorized as either “Academic Non-Faculty”, “Faculty”, “Industry / Non-Academic Research”, or “Clinical Practice or Teaching”. “Academic Non-Faculty” includes positions such as further postdoctoral research training. We also used previous T32 records and progress report data to determine trainee demographic information.

In the CV T32 Survey, all current and former trainees were asked to list the barriers and enablers they experienced to pursuing careers in academia from a predefined list of 21 barriers and 21 enablers. These barriers and enablers were identified and pooled from several previous reports on factors that influence early career scientists’ interest in pursuing academic careers [9–11]. To reduce bias, the order in which the barriers and enablers were listed was randomized. Respondents also had the option to specify up to three additional barriers or enablers. After selecting which barriers or enablers contributed to their decision to pursue a career in academia, respondents were asked to rank the barriers and enablers in order of greatest to least importance. Respondents could also write in their own responses, e.g. “Salary way too low,” even if those were redundant with existing choices. Note that not all cardiovascular T32 trainees participated in the CV T32 Survey. See supplementary information for the full survey (S5 File).

### Statistical analysis

Comparisons between different populations of trainees were performed using Chi-Square tests, with p-values < 0.05 considered significant. Bonferroni correction was used to adjust the p-value to correct for multiple comparisons.

### Materials, methods, and data availability

Source data from the OPA Dataset, the CV T32 Data, and the CV T32 Survey are included with this publication [S1–S4 Files] and on Dryad [12]. The R Markdown file used to create all three figures are available on Dryad. The survey instrument is included as supplementary material (S5 File).

## Results

Based on the data from our institution’s Office of Postdoctoral Affairs dataset, approximately 40% of health science-related postdocs attain faculty appointments after their training at our institution (Fig 1A). There is no significant difference in the proportion of trainees who become faculty compared to non-faculty between T32 Postdocs and Non-T32 Postdocs (p = 0.42, d.f. = 1). Conversely, former-T32 trainees are significantly more likely to attain appointments as primarily research faculty members, compared to other trainees (Fig 1B) (p = 0.002, d.f. = 1). Though limited by a small sample size, we also observed that compared to those that did not participate in a T32, a numerically higher proportion of postdocs who self-identified as underrepresented minorities went on to obtain a primarily research faculty position if they had participated in a T32. Of the 205 URM postdocs, 31.3% of the T32 trainees achieved such positions, compared to only 16.9% of URM trainees who participated in traditional post-doctoral fellowships. Although the outcomes of specifically URM trainees also reflected the relationship seen in comparing T32 and non-T32 trainees overall, and exhibited a larger effect size, this result was not significant at a threshold of 0.05 (p = 0.16 with Bonferroni correction.)

**Fig 2:**
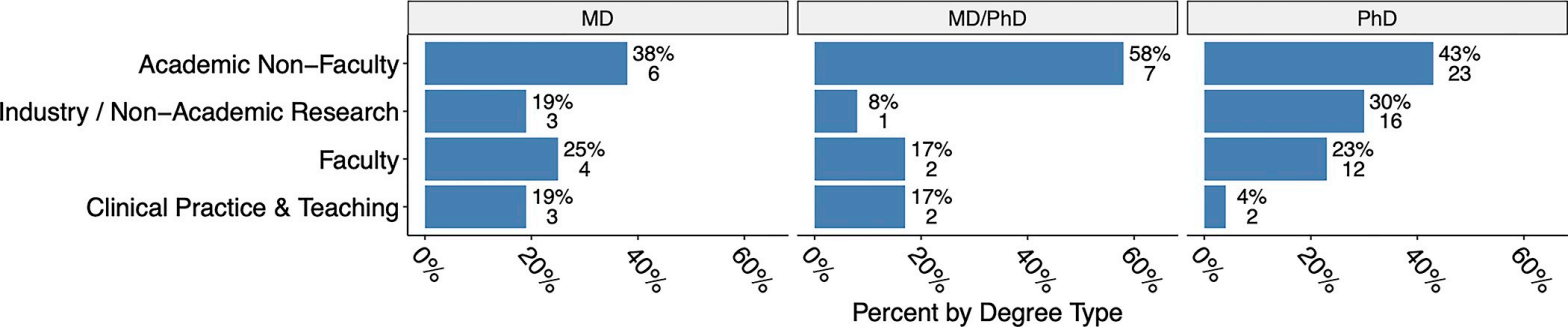
Distribution of T32 program trainees across different career sectors. Trainee data is categorized by their previous training background (MD, MD/PhD, PhD). Data from CV T32 Data, N = 81 T32 trainees.

We also assessed the distribution of T32 program trainees across different career sectors and given their graduate training background (e.g. MD, PhD, or MD/PhD). Trainees specifically in the three cardiovascular T32 programs had a similar distribution of proximal career trajectories, regardless of graduate degree type (Fig 2; p = 0.29, d.f. = 6). The highest proportion of T32 alumni from the programs had the proximal career stage of academic faculty; regardless of whether they received MD, PhD, or MD/PhD training. Notably, there was a low incidence of trainees entering clinical practice, regardless of MD, MD/PhD or PhD background. There was also no significant difference in the distribution of proximal career trajectories across cardiovascular T32 programs (p = 0.84, d.f. = 6, data available online [12]).

**Fig 3:**
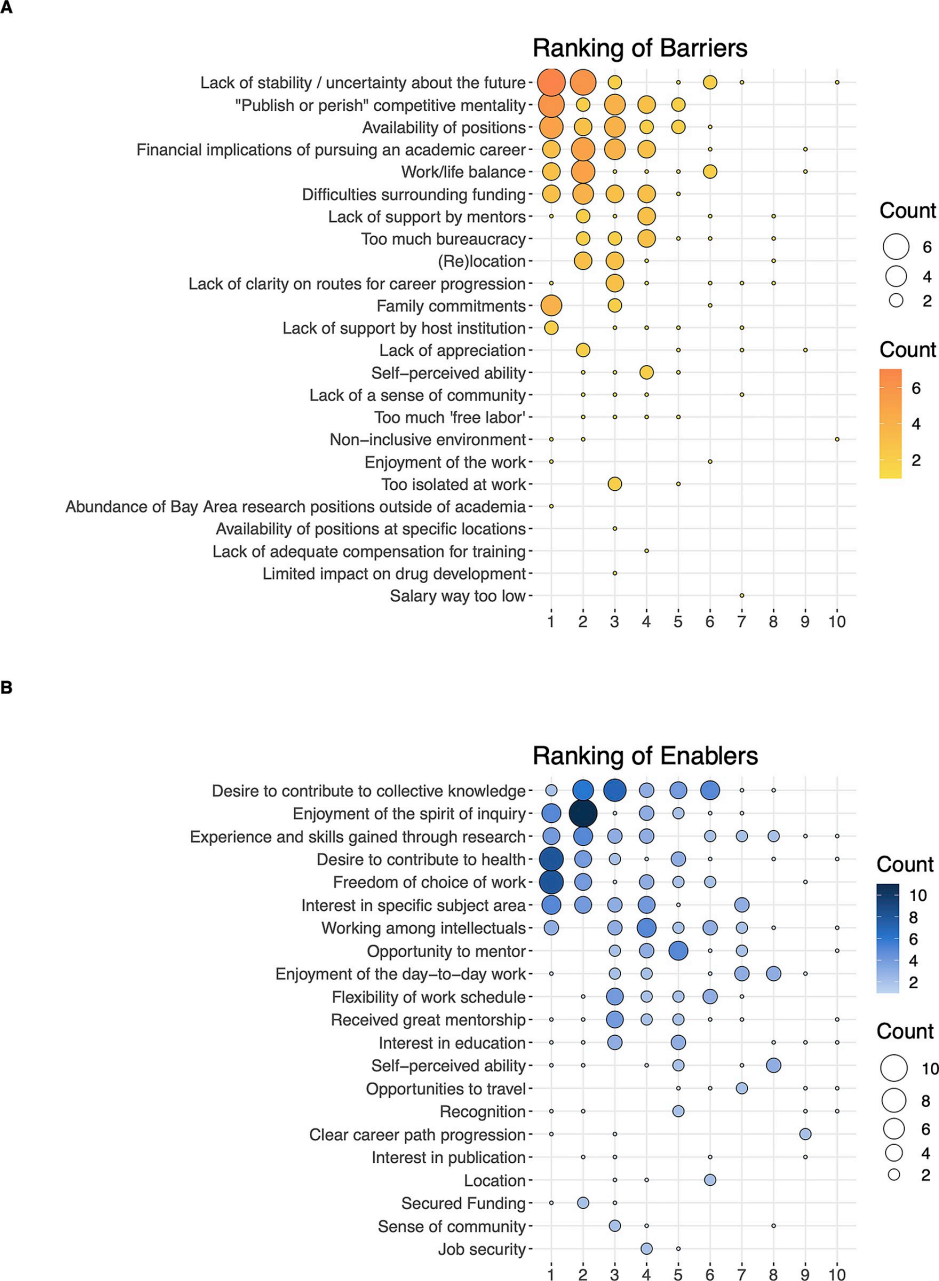
Ranking of barriers and enablers to pursuing careers in academia. Respondents could select any number of barriers or enablers and were then asked to rank them from 1^st^ (1, most significant barriers) to last. Barriers are listed in the upper panel, enablers in the lower panel. The size of the circles in this figure indicates the number of respondents who gave a specific barrier or enabler a specific rank. Data from CV T32 Survey, N = 49 survey responses.

We next surveyed trainees in the three cardiovascular T32 programs regarding the primary barriers and enablers to pursuing academic research careers. We counted the frequency that each barrier or enabler was ranked at a specific level of importance 1, 2, 3, etc). Fig 3 shows the number of times each barrier (upper panel) or enabler (lower panel) was ranked in each position. The size of the square corresponds to the frequency that barrier or enabler was chosen at that rank. The barriers and enablers are listed by decreasing frequency of selection. Cardiovascular T32 trainees identified the primary barriers to pursuing careers in academia as “Perceived Lack of Stability,” “Publish or Perish Competitive Mentality” and “Availability of Positions”. In contrast, they identified “Desire to Contribute to Collective Knowledge,” “Enjoyment of the Spirit of Inquiry,” and “Experience and Skills Gained Through Research” as the primary enablers. In general, respondents selected a greater number of enablers than barriers.

## Discussion

Maintaining the pipeline of the next generation of scientists and researchers has proven increasingly difficult. Even at a tier 1 research institution, less than half of the trained postdocs stay in academia, and less than a quarter of trainees attain primarily research faculty positions. Despite postdocs prevalent desire to contribute to collective knowledge, they describe being stymied in their pursuit of academic careers by numerous factors including uncertainty about their futures and the high degree of competition for faculty positions and publications. This suggests that more should be done to increase postdocs sense of security about their futures–that jobs will be available, and that their careers do not depend solely on generating high-impact publications.

### T32 training is correlated with obtaining primarily research faculty positions

One mechanism to enhance retainment in academic careers is to provide the infrastructure offered by programs like T32s that typically include formal grant writing programs, opportunities to network with visiting professors, guidance on establishing collaborations, public speaking, as well as other professional development opportunities. These opportunities may help reduce the barrier of the perceived lack of stability and make available positions more apparent. Additionally, structured training programs can increase postdoctoral trainees self-reported knowledge and growth and sense of independence and professional readiness [13].

At our university, T32 trainees ‘outperformed’ non-T32 trainees, with significantly higher rates of retention in specifically primarily research academic faculty positions. Primarily research faculty positions, in contrast to non-research (primarily teaching) faculty positions, require faculty devote significant effort to advancing scientific research projects. Faculty in these positions typically seek funding for research projects that contribute to their institutions’ total resources. The higher retention of T32 trainees in primarily research faculty positions was also true even amongst our domain-specific cardiovascular T32s, where high numbers of MDs turned down potentially more lucrative roles in industry or as practicing physicians to stay in academia.

### Barriers to academic research careers

At least in our sample, postdocs voiced strongest concerns about external factors: the uncertainty about finding a position and about their futures and a pervasive sense that their careers hinged on their publication track record. Postdocs were less inhibited about pursuing careers in academia by more personal concerns, such as a sense of community, mentorship, and the feeling that their work was appreciated. This suggests that more should be done to increase postdocs sense of security about their futures–that jobs will be available, and that their careers do not depend solely on generating high-impact publications. For this to be persuasive, hiring committees also need to look beyond applicants’ publication track records to other measures of success such as producing rigorous work, having creative ideas, and being an inclusive mentor, communicator, and educator. It is also worth noting that trainees were more strongly motivated to pursue careers in academia by more ‘lofty’ concepts such as the spirit of inquiry and contributing to collective knowledge, as opposed to, again, more tangible motivators such as the day-to-day work, opportunities to travel, and direct mentorship. This suggests that mentors, funding agencies, and institutions could be doing more to remind trainees of the value of their contributions, and to encourage them to embrace their curiosity and critical analysis.

### Relevance of structured training programs for trainees from underrepresented backgrounds

Our data further suggest that T32 training programs may be particularly valuable in supporting individuals from underrepresented backgrounds. We found that among URM trainees, a higher proportion of T32 trainees secured primary research faculty positions compared to non-T32 trainees; and indeed, the effect size was larger than in the postdoc population overall. However this difference was not statistically significant; possibly due to the low sample size of the URM data set. Future studies that specifically investigate the career outcomes of URM trainees in structured versus unstructured training programs are therefore warranted. Our results are consistent with a recent study investigating why underrepresented minorities and female postdocs in biomedical sciences may choose not to pursue careers in academic research that identified the most influential factors as ‘job prospects’ and ‘financial security’ [14]. This previously published study also identified that underrepresented minority postdocs had a very different experience of mentorship compared to well-represented postdocs and state they would benefit from additional support and specialized training. T32 mechanisms can fulfill those needs. Many T32 programs, including our three CV ones, implement a model of co-mentorship and provide supporting mentorship form the T32 program directors. This additional mentorship support could help buffer URM trainees from otherwise poor mentorship relationships with their primary research mentor. One solution for increasing the proportion of underrepresented minorities in faculty positions is to implement a new approach for increasing faculty diversity at the institutional level with ‘postdoc-to-tenure track conversion’ models [15]. These programs enable postdoctoral fellows to transition into a tenure-track role at the same institution they conduct their postdoc at.

### Limitations

Although this study provides insight into the motivations and challenges that trainees experience in pursuing academic careers, it is important to acknowledge that our data stems from a limited number of trainees who all experienced the same overall training environment at Stanford University. In addition, there may be an underlying factor that increases the likelihood both of trainees obtaining T32 awards and securing faculty positions, such as prior research history. For example, T32 programs may select candidates who are more competitive than other trainees due to their prior publication record. It is therefore possible that faculty review committees, who use similar selection criteria, are also more likely to offer positions to trainees based on the same criteria that helped secure their T32 awards. Another limitation of our study is that we were unable to verify the T32 status of all of the postdocs in the “Non-T32 Postdocs” group of our OPA dataset, and there may therefore still be a small percentage of T32 postdocs in this population (n = 54–108 out of 2,021). Because of the large size of this population and the relatively small proportion of T32 postdocs at Stanford (5%), the impact of T32-trainee presence in this population will be negligible and, if anything, would add noise to our results.

## Conclusion

Our results are consistent with the idea that dedicated training programs strengthen the pipeline of young scientists pursuing careers in academia as primarily research faculty. Recently, a study investigated the impact of NIH NRSA training programs such as T32s on career outcomes using NIH administrative records and found that receiving an NRSA fellowship significantly increased the probability of receiving subsequent research awards, indicating that federally funded fellowships such as T32 programs can promote the retention of scientists in the biomedical research workforce. [16]. In 2022, only 2.3% of the NIH budget went towards institutional training grants [17], yet this study and our data suggests that training programs are a valuable National investment in training motivated and qualified young researchers to pursue scientific discovery for the advancement of human health. However, even trainees in structured and supportive T32 training environments are often inhibited from pursuing academic careers by concerns about the uncertainty of their prospects and academia’s competitive culture. T32 training programs are a tool to increase the ability, motivation, and diversity of our academic workforce, but institutions also need to prioritize establishing transparent career ladders for faculty positions and look beyond publication metrics in their review of a faculty candidate’s academic merit.

## Supporting information

S1 Checklist STROBE statement—checklist of items that should be included in reports of *cross-sectional studies*. (PDF)

S1 File OPA data. (CSV)

S2 File CV T32 program data. (CSV)

S3 File CVI T32 survey data. (CSV)

S4 File Deidentified barriers and enablers. (XLSX)

S5 File Outcomes of T32 trainees survey. (DOCX)
