# Supplementary material for: Postdoctoral T32 training is correlated with obtaining an academic primarily research faculty position
Source: PLoS One. 2024 Jun 7;19(6):e0303792. doi: 10.1371/journal.pone.0303792 (PMC11161096; doi:10.1371/journal.pone.0303792)
Supplement: S5 File — (DOCX) [file pone.0303792.s006.docx]

Outcomes of T32 Trainees - Publication Version

Q1

In completing your postdoctoral or post-residency training, what were the biggest **barriers** to your pursuit of a career in academia?
If you are currently in training, please respond with your current thoughts.

- (Re)location (1)
- Availability of positions (2)
- Difficulties surrounding funding (3)
- Financial implications of pursuing an academic career (4)
- Family commitments (5)
- Enjoyment of the work (6)
- Lack of clarity on routes for career progression (7)
- Work/life balance (8)
- Lack of support by mentors (9)
- Lack of support by host institution (10)
- Contractual issues (11)
- Non-inclusive environment (12)
- Too much 'free labor' (13)
- Too isolated at work (14)
- Lack of stability / uncertainty about the future (15)
- Lack of appreciation (16)
- Lack of a sense of community (17)
- Too much bureaucracy (18)
- "Publish or perish" competitive mentality (19)
- Self-perceived ability (20)
- Abundance of Bay Area research positions outside of academia (21)
- Prefer to specify (22) ________________________________________________
- Prefer to specify (23) ________________________________________________
- Prefer to specify (24) ________________________________________________

| 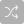 |
| --- |

Q2
In completing your postdoctoral or post-residency training, what were the biggest **enablers** to your pursuit of a career in academia?
If you are currently in training, please respond with your current thoughts.

- Secured funding (1)
- Received great mentorship (2)
- Experience and skills gained through research (3)
- Sense of community (4)
- Job security (5)
- Clear career path progression (6)
- Location (7)
- Interest in specific subject area (8)
- Desire to contribute to collective knowledge (9)
- Interest in education (10)
- Flexibility of work schedule (11)
- Opportunities to travel (12)
- Freedom of choice of work (13)
- Desire to contribute to health (14)
- Working among intellectuals (15)
- Recognition (16)
- Self-perceived ability (17)
- Interest in publication (18)
- Opportunity to mentor (19)
- Enjoyment of the day-to-day work (20)
- Enjoyment of spirit of inquiry (21)
- Prefer to specify (22) ________________________________________________
- Prefer to specify (23) ________________________________________________
- Prefer to specify (24) ________________________________________________

Carry Forward Selected Choices - Entered Text from "In completing your postdoctoral or post-residency training, what were the biggest barriers to your pursuit of a career in academia? If you are currently in training, please respond with your current thoughts."

|  |
| --- |

Q3
Please rank the **barriers** to pursuit of a career in academia in order of most important (top) to least important (bottom). You can drag and drop your selections.

______ (Re)location (1)

______ Availability of positions (2)

______ Difficulties surrounding funding (3)

______ Financial implications of pursuing an academic career (4)

______ Family commitments (5)

______ Enjoyment of the work (6)

______ Lack of clarity on routes for career progression (7)

______ Work/life balance (8)

______ Lack of support by mentors (9)

______ Lack of support by host institution (10)

______ Contractual issues (11)

______ Non-inclusive environment (12)

______ Too much 'free labor' (13)

______ Too isolated at work (14)

______ Lack of stability / uncertainty about the future (15)

______ Lack of appreciation (16)

______ Lack of a sense of community (17)

______ Too much bureaucracy (18)

______ "Publish or perish" competitive mentality (19)

______ Self-perceived ability (20)

______ Abundance of Bay Area research positions outside of academia (21)

______ Prefer to specify (22)

______ Prefer to specify (23)

______ Prefer to specify (24)

Carry Forward Selected Choices - Entered Text from "In completing your postdoctoral or post-residency training, what were the biggest enablers to your pursuit of a career in academia? If you are currently in training, please respond with your current thoughts."

|  |
| --- |

Q4

Please rank the **enablers** to pursuit of a career in academia in order of most important (top) to least important (bottom). You can drag and drop your selections.

______ Secured funding (1)

______ Received great mentorship (2)

______ Experience and skills gained through research (3)

______ Sense of community (4)

______ Job security (5)

______ Clear career path progression (6)

______ Location (7)

______ Interest in specific subject area (8)

______ Desire to contribute to collective knowledge (9)

______ Interest in education (10)

______ Flexibility of work schedule (11)

______ Opportunities to travel (12)

______ Freedom of choice of work (13)

______ Desire to contribute to health (14)

______ Working among intellectuals (15)

______ Recognition (16)

______ Self-perceived ability (17)

______ Interest in publication (18)

______ Opportunity to mentor (19)

______ Enjoyment of the day-to-day work (20)

______ Enjoyment of spirit of inquiry (21)

______ Prefer to specify (22)

______ Prefer to specify (23)

______ Prefer to specify (24)

| Page Break |  |
| --- | --- |

Q5
Could you briefly elaborate on "${Q15/ChoiceGroup/ChoiceWithLowestValue}" as a barrier to the pursuit of a career in academia?
If a second factor was a similarly strong influence, please elaborate on both.

________________________________________________________________

________________________________________________________________

________________________________________________________________

________________________________________________________________

________________________________________________________________

Q6

Could you briefly elaborate on "${Q16/ChoiceGroup/ChoiceWithLowestValue}" as an enabler to the pursuit of a career in academia?
 If a second factor was a similarly strong influence, please elaborate on both.

________________________________________________________________

________________________________________________________________

________________________________________________________________

________________________________________________________________

________________________________________________________________
